# Supplementary material for: Interpretable Drug-to-Drug Network Features for Predicting Adverse Drug Reactions
Source: Healthcare (Basel). 2023 Feb 17;11(4):610. doi: 10.3390/healthcare11040610 (PMC9957267; doi:10.3390/healthcare11040610)
Supplement: Supplementary file 1 [file healthcare-11-00610-s001.zip › healthcare-2152807-supplementary.pdf]

Supplementary Material S1:

Definition and calculation of the 10 network features included in this study are explained as below:

Degree centrality:

Degree centrality of a node in a network is defined as the number of adjacent edges connected to this node [24] and can be formalized as:

$$C(v) = \sum(e(vu))$$

Where e(vu) = 1 if there is a link between v and u and e(vu) = 0 if there is no link between v and u, for each node pair consisting of the focal node v and another node.

Weighted degree centrality:

For a weighted network, the weighted degree centrality of a focal node v is the sum of edge weights that the node v is related to [24] and this measure could be formalized as:

$$C(v) = \sum_{n=1}^{k}(w(vu))$$

Where u represents a surrounding node and the k is the number of such nodes. W(vu) represents the edge weights between node v and node u.

Eigenvector centrality:

The eigenvector centrality assesses the focal node's importance by considering its neighbors' importance as well [25]. We model the centrality of node as the sum of the centrality of neighboring nodes:

$$C(v) = \frac{1}{\lambda}\sum_{n=1}^{k}(\mathrm{C(u)})$$

Where λ is normalization constant, v is the node index to be calculated, u is the surrounding nodes of v and k is the number of surrounding nodes.

Closeness centrality:

As its name indicates, this measure means how close a node is to the network's all other nodes [25]. Accordingly by definition, a node is important if it has small shortest path lengths to all other nodes [26].

$$C(v) = \frac{1}{\sum_{u!=v}\mathrm{d(vu)}}$$

Where u is any other nodes than the focal v and d(vu) represents the shortest distance between v and u.

Clustering coefficient:

Clustering coefficient quantifies the degree to which the neighboring nodes around the focal one tend to cluster [27]. Intuitively, the more closely connected the neighboring nodes are, the more likely this focal node is important in this network. This measure is calculated with the below formula:

$$C(v) = \frac{\mathrm{N(e)}}{\binom{kv}{2}}$$

Where N(e) represents the number of edges connected to the focal bode v, and the denominator refers to the number of combinations of the neighboring nodes around v.

Betweenness centrality:

Betweenness centrality involves more global information of the network by considering the role that the focal node plays in the paths between any two given nodes [28]. This measure could be formalized as:

$$C(v) = \sum_{s!=v!=t}\frac{\mathrm{N}st(v)}{\mathrm{N}st}$$

Where Nst represents the total number of shortest paths from node s to node t and $Nst(v)$ represents the number of paths from node s to node t that go through node v.

Hub and Authority:

Hubs and authorities are a natural generalization of eigenvector centrality. The undirected, weighted graph with nodes labelled with their hub scores. Hubs are central nodes in this graph with respect to weighted eigenvector centrality. The details of these definitions and implementations could be found in Kleinberg Centrality [29].

Triangles:

This measure counts the number of triangles with the focal node and two other nodes. This could be expressed with the below formula:

$$C(v) = \sum e(vpq)$$

Where vpq are the three nodes within this network, and e(vpq) = 1 if these three nodes form a triangle (the three nodes are connected via three edges) and e(vpq) = 0 if these three nodes do not form a triangle.

PageRanks:

PageRanks bases the importance of a node on the link structure of the network and could be formalized as:

$$PR(v) = d \sum_{v|v \to u} \frac{PR(u)}{out(u)} + (1 - d)e(v)$$

Where e(v) is a fixed value called the personalization value and is given for each node v. To generate the rank of this node, (1−d)e(u) is added to the rank [30].

Reference

24. Opsahl, T., F. Agneessens, and J. Skvoretz, *Node centrality in weighted networks: Generalizing degree and shortest paths.* Social networks, 2010. **32**(3): p. 245-251.
25. Golbeck, J., *Chapter 3 - Network Structure and Measures*, in *Analyzing the Social Web*, J. Golbeck, Editor. 2013, Morgan Kaufmann: Boston. p. 25-44.
26. Freeman, L.C., *Centrality in social networks conceptual clarification.* Social networks, 1978. **1**(3): p. 215-239.
27. Holland, P.W. and S. Leinhardt, *Transitivity in structural models of small groups.* Comparative group studies, 1971. **2**(2): p. 107-124.
28. Barthélemy, M., *Betweenness centrality in large complex networks.* The European Physical Journal B, 2004. **38**(2): p. 163-168.
29. Kleinberg, J.M., *Authoritative sources in a hyperlinked environment.* Journal of the ACM (JACM), 1999. **46**(5): p. 604-632.
30. Brinkmeier, M., *PageRank revisited.* ACM Trans. Internet Technol., 2006. **6**(3): p. 282–301.
